# Right ventricular myocardial performance index as an early marker of cardiac dysfunction in systemic sclerosis

**DOI:** 10.3389/fmed.2025.1722534

**Published:** 2026-01-06

**Authors:** Emrah Kaya, Murat Taşçı, Uğur Karasu, Veli Çobankara, İlknur Kaya

**Affiliations:** 1 Department of Cardiology, Faculty of Medicine, Kütahya Health Sciences University, Kütahya, Türkiye; 2 Department of Rheumatology, Faculty of Medicine, Bolu Abant İzzet Baysal University, Bolu, Türkiye; 3 Department of Rheumatology, Faculty of Medicine, Pamukkale University, Denizli, Türkiye; 4 Department of Chest Diseases, Faculty of Medicine, Kütahya Health Sciences University, Kütahya, Türkiye

**Keywords:** Doppler echocardiography, interstitial lung disease, myocardial performance index, right ventricular (RV) dysfunction, subclinical cardiac involvement, systemic sclerosis

## Abstract

**Objectives:**

Systemic sclerosis (SSc) is associated with subclinical cardiac involvement often missed by conventional echocardiography. The right ventricular myocardial performance index (RV MPI), a Doppler-derived composite of systolic and diastolic function, has been proposed as an early marker of right ventricular (RV) dysfunction. This study was conducted to compare RV MPI between SSc patients and healthy controls and to determine its association with clinical and functional features of SSc.

**Methods:**

A cross-sectional study was performed in 60 patients with SSc and 83 age-matched healthy controls, all women. Comprehensive transthoracic echocardiography, including pulsed-wave Doppler of RV inflow and outflow, was used to calculate RV MPI as (IVCT + IVRT)/ET. Tricuspid annular plane systolic excursion (TAPSE) and the TAPSE/systolic pulmonary artery pressure (TAPSE/sPAP) ratio were recorded, together with disease duration, modified Rodnan skin score (mRSS) and pulmonary function tests (FVC, DLCO). Group comparisons and correlation analyses were conducted, and multivariable logistic regression and receiver operating characteristic (ROC) analyses were applied.

**Results:**

Left ventricular ejection fraction was similar between groups (median 60% vs. 61%), whereas RV MPI was found to be significantly higher in SSc than in controls (median 0.54 vs. 0.35, *p* < 0.001). Higher pulmonary artery systolic pressure, lower TAPSE and a reduced TAPSE/sPAP ratio were also observed in SSc (all *p* < 0.001). After adjustment for age and TAPSE/sPAP, RV MPI remained independently associated with SSc status. ROC analysis demonstrated excellent discrimination for SSc by RV MPI (area under the curve 0.92; threshold 0.47; sensitivity 78%, specificity 94%), whereas. TAPSE/sPAP showed only moderate discrimination. RV MPI was not significantly correlated with CRP, FVC, DLCO, mRSS or disease duration. In patients with interstitial lung disease, higher MPI values and more frequent DLCO < 80% were detected.

**Conclusion:**

RV MPI was shown to be significantly increased in SSc, even in the absence of overt cardiac symptoms or reduced left ventricular ejection fraction and remained independently associated with SSc. Together with reduced TAPSE and TAPSE/sPAP ratios, these findings indicate impaired RV–pulmonary arterial coupling. RV MPI therefore appears to be a simple and sensitive non-invasive parameter for the identification of cardiac involvement in SSc.

## Introduction

Systemic sclerosis (SSc) is a multisystem autoimmune condition that mostly affects the circulatory system and is characterized by fibrosis of the skin and internal organs. Cardiac manifestations occur frequently, with up to 80% of patients with SSc showing evidence of cardiac involvement in histological or imaging studies, often developing insidiously in the early stages while being clinically asymptomatic (1, 2). Right ventricular (RV) dysfunction is particularly pertinent in patients with SSc and often arises from pulmonary vascular disease or primary myocardial fibrosis. RV involvement and pulmonary arterial hypertension (PAH) are among the leading causes of morbidity and mortality in patients with SSc (3). Therefore, the early detection of cardiac dysfunction is crucial for identifying high-risk patients with SSc who may benefit from prompt intervention or closer monitoring. Transthoracic echocardiography is the mainstay of non-invasive cardiac evaluation in SSc and is recommended for the regular screening of RV function and PAH in these patients. However, conventional echocardiographic measurements may remain normal until late in the disease course, underscoring the need for more sensitive ventricular performance indices (3, 4).

A Doppler-derived indicator of global cardiac function, the myocardial performance index (MPI), often referred to as the Tei index, combines systolic and diastolic performance. The ratio of isovolumetric time to ejection time is used to compute MPI: MPI is calculated as (IVCT + IVRT)/ET, where ET is the ejection time, IVRT is the isovolumic relaxation time, and IVCT is the isovolumic contraction time (5). In practice, the MPI can be obtained by pulsed Doppler recordings of the inflow and outflow across the cardiac valves. The normal reference values for the RV MPI are approximately 0.28 ± 0.04 by conventional Doppler (6). Importantly, MPI is relatively independent of heart rate and blood pressure and reflects combined systolic/diastolic dysfunction. Prior studies in other populations have demonstrated that MPI is a sensitive early indicator of ventricular dysfunction and a prognostic marker. In the context of SSc, a few studies have suggested that even asymptomatic patients have an increased RV MPI, consistent with subtle RV impairment (7, 8). These findings raise the possibility that MPI could serve as an early quantitative marker of SSc-related cardiac dysfunction, even before conventional measures become abnormal in patients with SSc.

The primary objectives of this study were to comprehensively evaluate RV MPI in female patients with SSc compared to healthy female controls and to assess its association with clinical features. Specifically, we aimed to: (1) determine whether RV MPI is elevated in patients with SSc relative to controls; (2) examine correlations between RV MPI and disease parameters, including C-reactive protein (CRP) as a marker of inflammation, the modified Rodnan skin score as a measure of skin fibrosis, pulmonary function tests [forced vital capacity (FVC) and FEV1/FVC ratio], and SSc-specific autoantibodies; and (3) perform subgroup analyses of RV MPI in diffuse cutaneous vs. limited cutaneous SSc, stratified by antibody status [anti-topoisomerase I (Scl-70) vs. anti-centromere antibodies]. Additionally, we evaluated standard echocardiographic indices of RV function, including tricuspid annular plane systolic excursion (TAPSE) and the TAPSE/systolic pulmonary artery pressure (TAPSE/sPAP) ratio, and investigated their relationship with SSc status using multivariable modeling and ROC analysis. In this study, we investigated the utility of RV MPI as an early biomarker of cardiac involvement in patients with SSc and discussed its clinical implications in the context of disease monitoring and management.

## Materials and methods

### Study population

A cross-sectional comparative study was conducted involving 60 female patients with systemic sclerosis and 83 healthy female patients. All patients with SSc were adults who met ACR/EULAR criteria for SSc (limited or diffuse) and were enrolled from our institution’s rheumatology outpatient clinic. Key inclusion criteria for the SSc group were female gender (to eliminate gender-related cardiac differences) and a confirmed diagnosis of SSc. Patients with overt heart failure or significant valvular disease were excluded. All participants underwent echocardiographic measurements of tricuspid annular systolic excretion (TAPSE) as an index of right ventricular systolic function. TAPSE was recorded in the apical four-chamber view using M-mode, and values > 16 mm were considered normal (indicating preserved right ventricular systolic function). Individuals with decreased TAPSE (≤ 16 mm), indicating systolic right ventricular dysfunction, were excluded from the study. In total, 68 patients were initially screened; eight were excluded: five with TAPSE ≤ 16 mm and three with inadequate echocardiographic image quality.

The healthy control group comprised age-appropriate female volunteers without known cardiovascular or rheumatological diseases. Demographic data was recorded for all participants, and clinical data was collected for patients with SSc, including disease duration, clinical subtype, and organ involvement. None of the patients received targeted pulmonary arterial hypertension therapies or advanced heart failure medications at the time of echocardiography. Immunosuppressive treatment status was recorded, and no significant differences were observed between the clinical subgroups.

This study was approved by the Clinical Research Ethics Committee of the university (Approval No: 03/2019). All participants provided written informed consent in accordance with the Declaration of Helsinki.

### Echocardiographic assessment

Transthoracic echocardiography was performed in all participants using a Philips Affiniti CVx ultrasound machine (Philips Medical Systems, United States) equipped with an S5–1 MHz transducer. The patients were imaged in the left lateral decubitus position. Standard apical four-chamber (A4C) and parasternal short-axis (PSAX) views were obtained, with particular attention paid to the RV-focused view. Simpson’s biplane approach was used to assess the left ventricular ejection fraction (LVEF) in the echocardiogram’s apical view.

**FIGURE 1:**
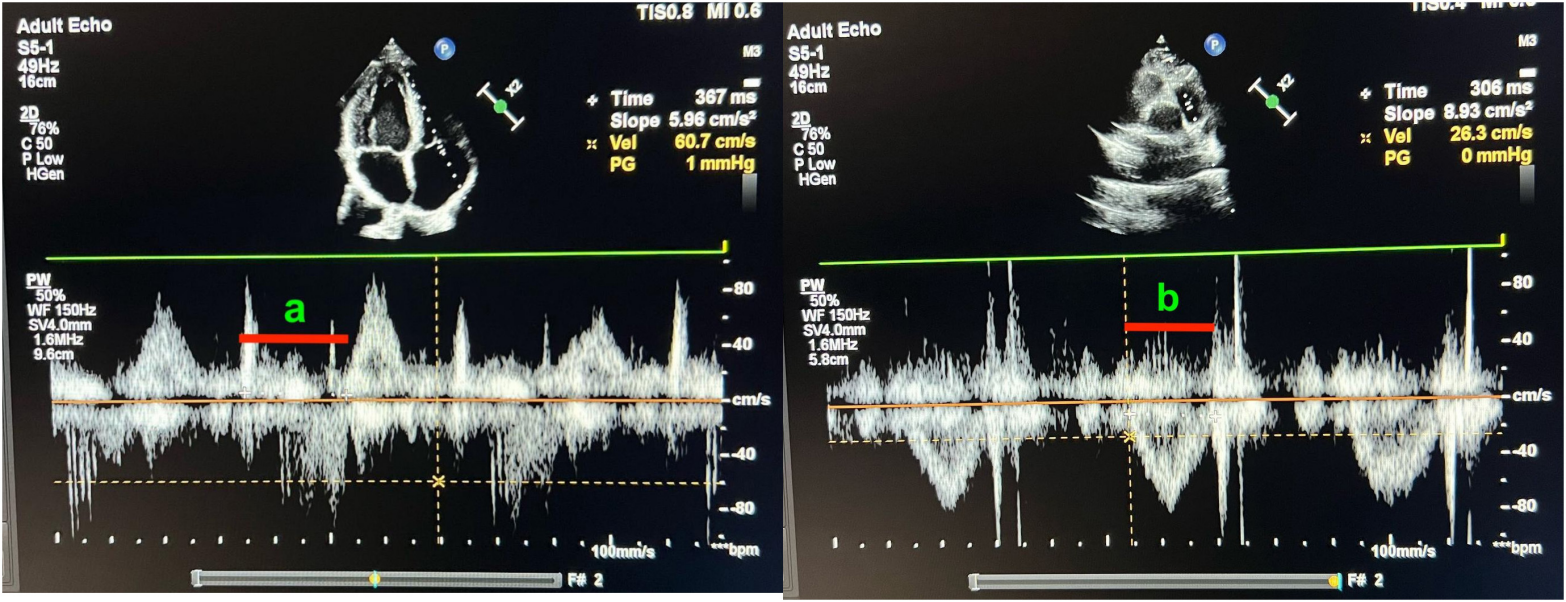
Measurement of right ventricular myocardial performance index (Tei Index) using pulsed-wave Doppler echocardiography. **(a)** Apical four-chamber view showing tricuspid inflow Doppler waveform. The interval labeled “a” corresponds to the total duration between tricuspid valve closure and opening (IVCT + ET + IVRT). **(b)** Parasternal short-axis view showing pulmonary outflow Doppler waveform. The interval labeled “b” indicates the ejection time (ET) of the right ventricle. The myocardial performance index (MPI) is calculated as (a - b)/b.

To evaluate global right ventricular function, the RV myocardial performance index (MPI) was measured using Doppler echocardiography and calculated as (a − b)/b. Here, a is the time interval from the end of RV inflow to the beginning of the next RV inflow and b is the RV ejection time. In practice, a adds the ejection time to the sum of the isovolumetric contraction and relaxation times, while b is the ejection time itself and is equivalent to MPI = (IVCT + IVRT)/ET. Doppler time intervals were obtained from the apical four-chamber (tricuspid inflow) and parasternal short-axis (RV outflow) views (shown in Figure 1). The “a” interval represents the total tricuspid valve opening-closing time, and the “b” interval represents the right ventricular ejection time.

RV outflow was recorded in the parasternal short-axis view with the sample volume in the pulmonary valve outflow tract. The difference between the total tricuspid closed-open time and pulmonic ejection time yielded the sum of the isovolumetric times. Each interval was measured across three consecutive beats and then averaged. All echocardiographic measurements were performed by an experienced cardiologist blinded to the participants’ clinical details. All time intervals and linear dimensions were measured and averaged across three consecutive cardiac cycles to minimize beat-to-beat variability. Measurements were performed according to the recommendations of the American Society of Echocardiography. MPI values were interpreted considering published normal ranges; right ventricular MPI > 0.40 measured by conventional Doppler was considered above the upper limit.

Additional RV parameters included TAPSE and the TAPSE/systolic pulmonary artery pressure (TAPSE/sPAP) ratio, calculated as TAPSE divided by PASP (mm/mmHg), as an echocardiographic measure of RV–pulmonary arterial (RV–PA) coupling. The estimated pulmonary artery systolic pressure (PASP), which was determined using the modified Bernoulli equation and the peak tricuspid regurgitation velocity, was one of the other echocardiographic measures of importance. We also qualitatively assessed regional wall motion abnormalities and the presence of pericardial effusion.

### Laboratory and clinical measurements

All patients with SSc underwent clinical assessment and laboratory testing on the same day as the echocardiography. Skin thickening was scored using the modified Rodnan skin score (mRSS), which assesses 17 body areas (total score range, 0–51). Serum C-reactive protein (CRP) levels were measured as a marker of systemic inflammation (mg/dL, by immunoturbidimetric assay). Pulmonary function tests were reviewed, including forced vital capacity (FVC% predicted) and the ratio of forced expiratory volume in 1 s to FVC (FEV1/FVC), to assess restrictive lung involvement. Diffusing capacity (DLCO% predicted) was available for a subset of patients. Autoantibody profiles were recorded from the clinical immunology laboratory: anti-topoisomerase I (Scl-70) and anti-centromere antibodies were determined by immunoassays, as were other antibodies (ANA, anti-RNA polymerase III, anti-U1 RNP, anti-SSA/Ro, anti-SSB/La, and rheumatoid factor) to characterize the cohort. Information on the current treatment was also recorded. None of the patients received targeted pulmonary arterial hypertension therapies or advanced heart failure medications at the time of echocardiography. Immunosuppressive therapy status (methotrexate, mycophenolate mofetil, and cyclophosphamide) was documented, and no significant differences in treatment distribution were observed between the clinical subgroups. For health controls, basic demographic data and a screening physical examination were obtained, but laboratory tests were not performed, except to ensure normal baseline health. As this was a cross-sectional study, no longitudinal follow-up data was available for the outcome analysis.

### Statistical analysis

Data were analyzed using SPSS 26.0 (IBM Corp.) and are presented as mean ± standard deviation (SD) for continuous variables or counts (percentages) for categorical variables. The distribution of continuous variables was assessed for normality using the Shapiro–Wilk test. For between-group comparisons of SSc patients and controls, an unpaired Student’s *t*-test was used for normally distributed variables, and the Mann–Whitney U test was used for non-normal distributions. Categorical variables were compared using the chi-square or Fisher’s exact test, as appropriate.

Correlations between RV MPI and clinical/laboratory parameters were evaluated using Pearson’s correlation coefficient if both variables were approximately normally distributed, or Spearman’s rank correlation for non-parametric analysis. Analogous correlation analyses were performed for the TAPSE/sPAP ratio. In the SSc subgroup analysis, we compared the diffuse and limited cutaneous subsets using independent t- and Mann–Whitney tests for MPI and other continuous measures.

To further evaluate the discriminatory performance of RV MPI and TAPSE/sPAP in distinguishing SSc patients from healthy controls, receiver operating characteristic (ROC) curve analyses were performed and the area under the curve (AUC) was calculated. Optimal cut-off values to maximize sensitivity and specificity were determined using the Youden index.

Multivariate logistic regression analysis was used to determine whether RV MPI was independently associated with SSc status after adjusting for potential confounders such as age and TAPSE/sPAP. Odds ratios (OR) and 95% confidence intervals (CI) were calculated. For all analyses, a two-sided *p* < 0.05 was considered statistically significant. Missing data, including DLCO measurements (*n* = 10), were handled using listwise exclusion in the respective analyses.

## Results

Sixty female patients with SSc and 83 age-matched healthy female controls were included in this study. The mean age of the SSc group was 54 years (IQR: 43–62 years), while the control group had a median age of 48 years (IQR: 37–56 years). Among patients with SSc, the median duration since diagnosis was 13 years (IQR: 10–16). The median score on the modified Rodnan skin score, which measures cutaneous involvement, was 10 (IQR: 5–17). Raynaud’s phenomenon was reported in 83.3 of patients, and 46.7% exhibited interstitial lung disease (ILD). High-resolution computed tomography (HRCT) revealed ground-glass opacities in 41.7% of the cohort and honeycomb patterns in 8.3% of the cohort. Gastrointestinal involvement and secondary Sjögren’s syndrome were observed in 43.3 and 46.7% of patients, respectively. Digital ulcers were present in 23.3% of patients and telangiectasia in 43.3% of patients. Pulmonary function tests revealed a median FVC of 90% (IQR: 84–102) and mean FEV1/FVC ratio of 85 ± 6%. The diffusing capacity for carbon monoxide (DLCO) was available for 50 patients, with a median of 78% (IQR: 67–96). The median 6-min walk distance was 420 m (IQR: 360–450 m). Autoantibody testing showed positivity for anti-Scl-70 in 28.3%, anti-centromere antibodies in 25.0%, anti-SSA in 20.0%, anti-SSB in 10.0%, and anti-U1 RNP in 10.0% of patients (Table 1).

**TABLE 1:** Baseline clinical, functional, and serologic characteristics of systemic sclerosis patients.

Characteristic	SSc patients (*n* = 60)
Height, cm	158 ± 7
Weight, kg, mean ± SD	66 ± 12
Disease duration, years, median [IQR]	13 [10–16]
Modified Rodnan skin score, median [IQR]	10 [5–17]
Raynaud phenomenon, n (%)	50 (83.3%)
Digital ulcers, n (%)	14 (23.3%)
Telangiectasia, n (%)	26 (43.3%)
Calcinosis, n (%)	3 (5.0%)
Interstitial lung disease (ILD), n (%)	28 (46.7%)
Ground-glass opacities on HRCT, n (%)	25 (41.7%)
Honeycombing on HRCT, n (%)	5 (8.3%)
GI involvement, n (%)	26 (43.3%)
Secondary Sjögren’s syndrome, n (%)	28 (46.7%)
History of arthritis, n (%)	5 (8.3%)
Active arthritis, n (%)	3 (5.0%)
FVC,% predicted, median [IQR]	90 [84–102]
FEV1/FVC,%, mean ± SD	85 ± 6
DLCO,% predicted †, median [IQR]	78 [67–96]
6-minute walk distance, m, median [IQR]	420 [360–450]
Hemoglobin, g/dL, mean ± SD	12.5 ± 1.4
WBC, × 103/μL, median [IQR]	7.2 [5.5–8.9]
Platelets, × 103/μL, mean ± SD	279 ± 85
ESR, mm/hour, median [IQR]	22 [14–37]
CRP, mg/L, median [IQR]	1.4 [0.9–4.0]
RF positive, n (%)	8 (13.3%)
Anti-Scl-70 positive, n (%)	17 (28.3%)
Anti-centromere positive, n (%)	15 (25.0%)
Anti-SSA positive, n (%)	12 (20.0%)
Anti-SSB positive, n (%)	6 (10.0%)
Anti-U1 RNP positive, n (%)	6 (10.0%)

SSc, systemic sclerosis; HRCT, high-resolution computed tomography; FVC, forced vital capacity; FEV1/FVC,%,, ratio of forced expiratory volume in 1 s to forced vital capacity; DLCO, diffusing capacity for carbon monoxide († was available in 50 patients); ESR, erythrocyte sedimentation rate; CRP, C-reactive protein.

LVEF was preserved in both groups, and no statistically significant difference was found [median 60% (IQR: 59–64) in the SSc group and 61% (IQR: 60–64) in the control group; *p* = 0.64). Compared with the control group, PASP was significantly higher in SSc patients (median 28 mmHg (IQR: 17–34) vs. 18 mmHg (IQR: 15–24], *p* < 0.001]. As shown in the updated group comparison (Table 2), significantly lower TAPSE values [median 18.0 mm (IQR: 17.0–19.0) vs. 19.0 mm (IQR: 18.0–21.0), *p* < 0.001] and reduced TAPSE/sPAP ratio [median 0.65 (IQR: 0.55–1.13]) vs. 1.11 (IQR: 0.82–1.38), *p* < 0.001] were also observed in SSc patients, consistent with impaired right ventriculoarterial coupling and systolic performance. RV MPI is shown to be significantly higher in the SSc group [median 0.54 (IQR: 0.47–0.59)] compared to the control group [0.35 (IQR: 0.28–0.39)] (*p* < 0.001) (Table 2).

**TABLE 2:** Comparison of key echocardiographic and demographic parameters between SSc patients and healthy controls.

Characteristic	SSc patients (*n* = 60)	Healthy controls (*n* = 83)	*p*-value^1^
Age, years	54.5 [43.0–62.2]	48.0 [37.0–55.5]	0.006
LVEF,%	60.0 [59.0–64.0]	61.0 [60.0–64.0]	0.635
PASP, mmHg	28.0 [16.8–34.2]	18.0 [15.0–23.5]	<0.001
TAPSE, mm	18.0 [17.0–19.0]	19.0 [18.0–21.0]	<0.001
TAPSE/sPAP, mm/mmHg	0.65 [0.55–1.13]	1.11 [0.82–1.38]	<0.001
RVMPI	0.54 [0.47–0.59]	0.35 [0.28–0.39]	<0.001

Values are given as median [interquartile range].

^1^Mann–Whitney U test. Comparison of age and key echocardiographic parameters between systemic sclerosis (SSc) patients and healthy controls. RV MPI indicates right ventricular myocardial performance index; TAPSE/sPAP denotes the tricuspid annular plane systolic excursion to systolic pulmonary arterial pressure ratio (an index of RV–PA coupling).

A comparison of the key echocardiographic and demographic parameters between the two groups is presented in Table 2.

RV MPI did not show a statistically significant correlation with key inflammatory markers, disease duration, or extent of skin involvement in the primary correlation analysis. Similarly, no correlation was observed between RV MPI and pulmonary function parameters or 6-min walk distance in the overall SSc cohort. A marginal association was observed between MPI and Raynaud’s phenomenon (*r* = 0.24, *p* = 0.06), although it was not statistically significant. The presence of honeycombing on high-resolution CT exhibited a modest inverse correlation with MPI (*r* = −0.26, *p* = 0.04). The correlation analysis between RV MPI and the clinical, functional, and serological parameters in the SSc group is presented in Table 3.

**TABLE 3:** Correlation of right ventricular myocardial performance index (RV MPI) with clinical, functional, and serologic parameters in SSc patients.

Parameter	*R*	*p*-value	Test
**Demographic**			
Age (years)	0.00	0.99	*
Height (cm)	–0.08	0.54	#
Weight (kg)	0.20	0.11	#
**Clinical**			
Disease duration (years)	–0.21	0.11	*
Modified Rodnan skin score (0–51)	+ 0.30	0.03	*
Raynaud phenomenon (present)	0.24	0.06	‡
Digital ulcers (present)	0.07	0.61	‡
Telangiectasia (present)	0.16	0.23	‡
Interstitial lung disease (present)	0.00	0.98	‡
Ground-glass opacities (present)	0.06	0.67	‡
Honeycombing (present)	–0.26	0.04	‡
GI involvement (present)	0.13	0.31	‡
6-min walk distance (m)	–0.08	0.57	*
**Pulmonary function**			
FVC (% predicted)	–0.05	0.14	*
FEV1/FVC (%)	–0.04	0.75	#
DLCO (% predicted)	0.09	0.54	#
**Laboratory**			
Hemoglobin (g/dL)	0.09	0.49	#
WBC (× 10^3^/μL)	–0.22	0.09	*
Platelets (× 10^3^/μL)	–0.15	0.26	#
ESR (mm/hour)	0.03	0.80	*
CRP (mg/L)	–0.13	0.33	*
Rheumatoid factor (IU/mL)	0.09	0.48	*
**Serologic**			
Anti-Scl-70 antibody (positive)	–0.02	0.85	‡
Anti-centromere antibody (positive)	0.20	0.11	‡
Anti-SSA antibody (positive)	–0.02	0.85	‡
Anti-SSB antibody (positive)	0.09	0.51	‡
Anti-U1 RNP antibody (positive)	–0.08	0.54	‡

*Spearman, #Pearson, ‡Point-biserial. Statistical significance was set at *p* < 0.05. SSc, systemic sclerosis; RV MPI, right ventricular myocardial performance index; FVC, forced vital capacity; FEV1, forced expiratory volume in 1 s; DLCO, diffusing capacity of the lungs for carbon monoxide; WBC, white blood cell count; ESR, erythrocyte sedimentation rate; CRP, C-reactive protein; RF, rheumatoid factor; GI, gastrointestinal.

RV MPI was significantly higher in patients with ILD than in patients without ILD (0.58 ± 0.12 vs. 0.50 ± 0.13, *p* = 0.01). Significantly higher MPI values were seen in patients with DLCO < 80% (0.59 ± 0.11 vs. 0.49 ± 0.12, *p* = 0.003). Although MPI values in patients with mRSS ≥ 15 were numerically higher than in those with mRSS < 15 (0.55 ± 0.09 vs. 0.50 ± 0.11), the difference did not reach statistical significance (*p* = 0.045 in some comparisons, trend level in others). Comparisons between anti-Scl-70 positive and anti-centromere positive patients revealed no significant difference in MPI (0.55 ± 0.13 vs. 0.52 ± 0.14, *p* = 0.45). Subgroup comparisons of RV MPI values across the clinically relevant systemic sclerosis categories are presented in Table 4.

**TABLE 4:** Right ventricular myocardial performance index (RV MPI) in systemic sclerosis (SSc) patient subgroups.

Subgroup comparison	Group 1 (n)	RV MPI	Group 2 (n)	RV MPI	*p*-value
Scl-70 antibody positive vs. Centromere antibody positive	17	0.55 ± 0.13	15	0.52 ± 0.14	0.45
Interstitial lung disease, present vs. absent	28	0.58 ± 0.12	32	0.50 ± 0.13	0.01
DLCO < 80% predicted vs. ≥ 80% predicted	25	0.59 ± 0.11	25	0.49 ± 0.12	0.003
Modified Rodnan skin score ≥ 15 vs. < 15	24	0.55 ± 0.09	36	0.50 ± 0.11	0.045
Anti-SSA antibody positive vs. negative	12	0.51 ± 0.10	48	0.53 ± 0.10	0.73
Honeycombing on HRCT present vs. absent	5	0.60 [0.58–0.65]	55	0.52 [0.47–0.58]	0.08

Student’s *t*-test, Mann–Whitney U. SSc, systemic sclerosis; ILD, interstitial lung disease; HRCT, high-resolution computed tomography; DLCO, diffusing capacity for carbon monoxide; SSA, anti–Sjögren’s-syndrome-related antigen A; MPI, myocardial performance index. Subgroup definitions: Group 1: Scl-70 positive patients (*n* = 17); Group 2: Centromere antibody positive patients (*n* = 15). Group 1: Patients with interstitial lung disease (ILD) (*n* = 28); Group 2: Patients without ILD (*n* = 32). Group 1: Patients with DLCO < 80% predicted (*n* = 25); Group 2: Patients with DLCO ≥ 80% predicted (*n* = 25). Group 1: Patients with a modified Rodnan skin score ≥ 15 (*n* = 24); Group 2: Patients with a Rodnan score < 15 (*n* = 36). Group 1: Anti-SSA antibody positive patients (*n* = 12); Group 2: Anti-SSA antibody negative patients (*n* = 48). Group 1: Patients with honeycombing on HRCT (*n* = 5); Group 2: Patients without honeycombing (*n* = 55).

In a separate correlation analysis, RV MPI demonstrated statistically significant associations with several clinical and functional parameters in earlier exploratory models; higher RV MPI values were moderately correlated with reduced DLCO, lower FVC, and increased PASP, and showed a weak-to-moderate positive correlation with mRSS (Table 5). These relationships supported the concept that global RV dysfunction is linked with pulmonary involvement and more extensive fibrotic burden in SSc.

**TABLE 5:** Correlation between right ventricular myocardial performance index (RV MPI) and clinical/functional parameters in systemic sclerosis patients.

Parameter	Spearman’s ρ	*p*-value
DLCO (% predicted)	–0.40	0.004
FVC (% predicted)	–0.32	0.02
PASP (mmHg)	+0.38	0.006
mRSS (Modified Rodnan Skin Score)	+0.30	0.03
Age (years)	+0.05	0.71
Raynaud phenomenon (present vs. absent)	+0.28	0.045
Anti-Scl-70 (positive vs. negative)	+0.33	0.02
Anti-centromere (positive vs. negative)	+0.20	0.08

Spearman’s rank correlation coefficient (ρ). Statistical significance threshold was set at *p* < 0.05. RV MPI, right ventricular myocardial performance index; DLCO, diffusing capacity of the lungs for carbon monoxide; FVC, forced vital capacity; PASP, pulmonary artery systolic pressure; mRSS, modified Rodnan skin score.

Within the SSc cohort, the TAPSE/sPAP ratio showed a strong and inversely proportional relationship with PASP (Spearman ρ≈−0.99, *p* < 0.001), as expected given its mathematical definition, and a positive relationship with TAPSE (ρ≈ 0.29, *p* ≈ 0.025). A trend-level negative relationship was observed between TAPSE/sPAP and age (ρ≈−0.25, *p* ≈ 0.051), suggesting a slightly worsening of RV-PA coupling with advancing age.

### Multivariable logistic regression and ROC analyses

In a multivariable logistic regression model including RV MPI, TAPSE/sPAP, and age, the presence of SSc (compared with control) served as the dependent variable. The model showed good fit overall (Nagelkerke pseudo R^2^ ≈ 0.53, odds ratio *p* < 0.001). After adjusting for TAPSE/sPAP and age, RV MPI remained independently associated with SSc status: each 0.1-unit increase in MPI was associated with an approximately 9.4-fold higher odds of SSc (OR 9.37, 95% CI 4.55–19.29, *p* < 0.001). The TAPSE/sPAP ratio trended toward an inverse association with SSc (OR 0.31, 95% CI 0.08–1.23, *p* = 0.096), whereas age was not independently associated with SSc in this model (OR 1.02/year, 95% CI 0.98–1.07, *p* = 0.34) (Table 6).

**TABLE 6:** Multivariable logistic regression model for discrimination between SSc patients and healthy controls dependent variable: presence of SSc (1 = SSc, 0 = control).

Variable	Adjusted OR	95% CI	*p*-value^1^
RV MPI (per 0.1-unit increase)	9.37	4.55–19.29	< 0.001
TAPSE/sPAP (per 1 mm/mmHg increase)	0.31	0.08–1.23	0.096
Age (per 1-year increase)	1.02	0.98–1.07	0.344

Model fit (Logit): *n* = 143; Nagelkerke pseudo *R*^2^ ≈ 0.53; likelihood ratio *p* < 0.001.

^1^Wald test. Multivariable logistic regression analysis evaluating the association of RV MPI, TAPSE/sPAP ratio, and age with the presence of systemic sclerosis (SSc) compared with healthy controls.

In ROC analysis, RV MPI demonstrated excellent discrimination ability in distinguishing SSc patients from controls, with an AUC of 0.92. The optimal cutoff value for RV MPI of 0.47 yielded a sensitivity of 78% and a specificity of 94%. The TAPSE/sPAP ratio demonstrated moderate discrimination power, with an AUC of 0.71; A cutoff value of ≤ 0.67 mm/mmHg provided 55% sensitivity and 83% specificity for SSc (Table 7).

**TABLE 7:** ROC analysis of RV MPI and TAPSE/sPAP for discrimination between SSc patients and healthy controls.

Marker	AUC	Optimal cutoff^1^	Sensitivity (%)	Specificity (%)
RV MPI	0.92	0.47	78	94
TAPSE/sPAP	0.71	0.67 mm/mmHg^2^	55	83

^1^Optimal cutoff determined by maximization of the Youden index.

^2^For TAPSE/sPAP, the discriminative threshold corresponds to TAPSE/sPAP ≤ 0.67 mm/mmHg indicating SSc. Receiver operating characteristic (ROC) analysis of RV MPI and TAPSE/sPAP ratio for differentiating systemic sclerosis (SSc) patients from healthy controls.

**FIGURE 2:**
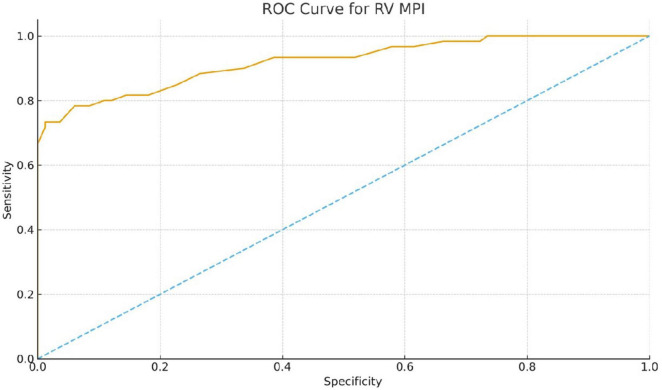
Receiver operating characteristic (ROC) curve of right ventricular myocardial performance index (RV MPI) for differentiating systemic sclerosis (SSc) patients from healthy controls. The RV MPI showed excellent discriminative ability, with an area under the curve (AUC) of 0.92. Using a cutoff value of 0.47, RV MPI identified SSc with 78% sensitivity and 94% specificity.

To assess the diagnostic ability of classic systemic sclerotic parameters in predicting high right ventricular MPI (defined as > 0.40), ROC curve analyses were also performed for DLCO and mRSS. DLCO had a limited discriminatory value with an AUC of 0.52. The optimal cutoff value of DLCO ≤ 107.7% provided high sensitivity (1.00) but low specificity (0.13). Lower DLCO levels tended to be associated with a higher MPI, but DLCO alone was not sufficient to reliably identify patients with subclinical right ventricular dysfunction (Figure 2).

**FIGURE 3:**
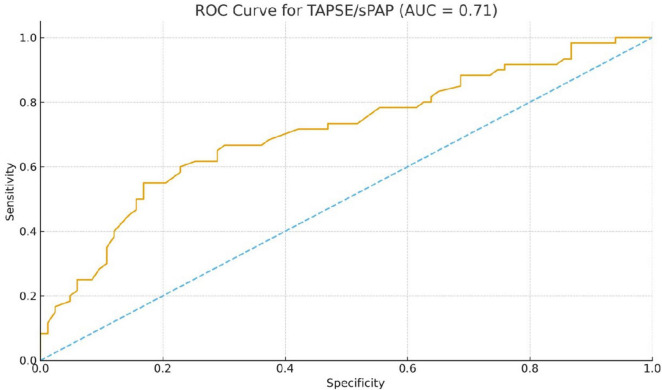
Receiver operating characteristic (ROC) curve of the tricuspid annular plane systolic excursion to systolic pulmonary arterial pressure (TAPSE/sPAP) ratio for differentiating systemic sclerosis (SSc) patients from healthy controls. The TAPSE/sPAP ratio demonstrated moderate discriminative performance, with an area under the curve (AUC) of 0.71. A threshold of 0.67 mm/mmHg yielded 55% sensitivity and 83% specificity for the identification of SSc.

mRSS did not demonstrate diagnostic accuracy in detecting high MPI levels, with an AUC of 0.44. Although the optimal cutoff value of mRSS ≥ 23.2 achieved 100% specificity, its sensitivity was only 10%, confirming that severe skin fibrosis is not a sensitive marker of early cardiac involvement in SSc (Figure 3).

## Discussion

In this study, we found that patients with SSc had a significantly elevated RV MPI compared to healthy controls, despite having preserved LVEF. This finding is consistent with the growing body of literature indicating subclinical cardiac dysfunction in patients with SSc. For example, Karna et al. reported markedly higher RV MPI values in SSc patients versus controls (0.54 vs. 0.35, *p* < 0.001) (9), and Ciurzyński et al. similarly observed an increased RV Tei index in SSc (mean 0.35) compared to controls (0.29) (10). These concordant results reinforce that global RV performance is impaired in SSc, even when conventional measures of LV systolic function remain normal (11). Notably, the lack of difference in LVEF between the SSc and control groups in our cohort aligns with prior studies showing that standard LV function can be preserved, whereas RV function is subtly compromised. Our findings are in line with recent syntheses showing that even apparently asymptomatic patients with connective tissue disease exhibit early biventricular involvement on imaging, which carries prognostic implications (8, 12, 13).

We also found no significant differences in RV MPI between the diffuse and limited SSc subtypes. This finding is consistent with earlier reports that echocardiographic indices of cardiac function do not consistently differ between SSc subtypes. For instance, Karna et al. noted no significant variation in key echocardiographic parameters between diffuse and limited SSc, suggesting that both subtypes are susceptible to similar degrees of subclinical myocardial impairment (9). However, our subgroup analyses revealed that certain high-risk clinical features correlated with worse RV MPI: patients with evidence of ILD had higher MPI than those without, those with more advanced skin fibrosis (Rodnan skin score ≥ 15) had higher MPI than those with mild skin involvement, and patients with reduced DLCO had higher MPI than those with normal DLCO. These observations correlate with the results of Huez et al. and others that pulmonary involvement in SSc can aggravate RV dysfunction. Huez et al. demonstrated that many SSc patients exhibit isolated RV dysfunction at rest and an inappropriate exercise-induced pulmonary pressure response, implicating “latent” pulmonary vascular disease as a driver of early RV impairment (14). Consistently, our SSc patients with ILD and low DLCO, features often associated with pulmonary vascular involvement or hypoxia, had a worse RV MPI. In contrast, we observed no significant correlation between RV MPI and CRP, FVC, or disease duration, indicating that the degree of RV dysfunction did not simply mirror general inflammation, lung capacity, or time since diagnosis. This suggests that subclinical cardiac involvement in SSc can occur independently of overt inflammation or long disease duration, reinforcing the need for dedicated cardiac evaluation in these patients, regardless of systemic inflammatory markers.

Our findings underscore the clinical value of RV MPI as a sensitive marker of early cardiac involvement in patients with SSc. MPI is an easily obtainable Doppler measure that integrates both systolic and diastolic functions of the ventricles. In the context of SSc, where overt cardiac symptoms are often absent until late in the disease course, an elevated RV MPI can serve as an early warning sign of “occult” myocardial dysfunction (8). The usefulness of MPI in the diagnosis of connective tissue disorders has been shown in earlier research. A recent systematic review and meta-analysis of 22 studies found that patients with autoimmune connective tissue diseases, including SSc, had significantly higher MPI values than controls and concluded that the MPI has the potential for early detection and management of cardiac dysfunction in this population (12). Clinically, this means that even if an SSc patient has a normal LVEF and no cardiac symptoms, a prolonged RV MPI might prompt closer surveillance or further testing for cardiac involvement. In our cohort, some patients with SSc and elevated MPI had no overt signs of heart failure or pulmonary hypertension; however, this subtle dysfunction could present future problems. Therefore, using RV MPI in routine echocardiographic evaluations of patients with SSc could improve the early identification of patients at risk of developing heart failure or pulmonary hypertension. Importantly, several studies have linked abnormal MPI to a worse prognosis in SSc. For example, the global Tei index correlates with lower survival rates in patients with SSc, emphasizing that detecting an elevated MPI is not only of academic interest but may also have direct prognostic significance (15). Thus, RV MPI can be integrated into risk stratification models for SSc. Patients with SSc with a high RV MPI may require more aggressive monitoring for cardiopulmonary complications than an SSc patient with normal MPI. In summary, our study adds to the evidence that RV MPI is a sensitive and clinically meaningful index of subclinical cardiac dysfunction in SSc, which is simple to measure and may fill an important gap in current SSc care by identifying early RV impairment before irreversible damage occurs (12).

The observed RV impairment in patients with SSc likely reflects a multifactorial pathophysiology. A key mechanism is myocardial fibrosis caused by SSc-related microvascular disease. SSc is characterized by diffuse microangiopathy and vascular dysfunction. In the heart, repeated episodes of microvascular ischemia can lead to patchy myocardial fibrosis and scarring of the ventricles. This process can stiffen the myocardium and subtly impair contraction and relaxation, elevating the MPI even if the overall EF remains normal. Such fibrosis-related diastolic dysfunction was documented by Ciurzyński et al., who found a high prevalence of abnormal ventricular filling patterns in patients with SSc (10). Moreover, left ventricular diastolic dysfunction has been independently associated with mortality in SSc, as demonstrated by Tennøe et al., who emphasized the prognostic relevance of early myocardial impairment (16). Our finding that RV MPI elevation did not correlate with CRP levels suggests that chronic fibrotic remodeling, rather than acute inflammation, may be the dominant contributor to RV dysfunction. Intrinsic myocardial involvement is another factor that can directly affect the myocardium via immune-mediated injury and collagen deposition in the heart muscle and conduction system. Over time, this can reduce myocardial compliance and contractility. Notably, we found that patients with more severe skin fibrosis had worse RV MPI, suggesting that a more aggressive fibrotic phenotype may extend systemically to the heart. However, even limited SSc patients can have significant cardiac fibrosis in autopsy studies, so clinical skin extent is not a perfect predictor; hence, there is a lack of MPI difference between diffuse and limited subsets in our cohort and in previous reports (13, 17, 18).

In addition, pulmonary vascular disease and RV afterload play critical roles in the cardiac dysfunction associated with SSc. SSc can lead to pulmonary arterial hypertension and/or pulmonary fibrosis, both of which increase pressure on the RV. Chronic elevation of RV afterload causes RV hypertrophy and dysfunction. In our study, patients with SSc with ILD and those with low DLCO had significantly higher RV MPI, implicating pulmonary involvement as a driver of RV impairment in these patients. Even before frank PAH is diagnosed, many SSc patients have “latent” pulmonary hypertension—subtle pulmonary vascular changes that only manifest under stress. Huez et al. showed that SSc patients with normal resting pulmonary pressures often exhibit an abnormal rise in pulmonary artery pressure during exercise, along with reduced RV diastolic function (14). They posited that this latent pulmonary hypertension leads to early RV diastolic dysfunction in SSc, which can be unmasked by exercise or Doppler indices, such as the MPI. Our data support this concept; the association of high MPI with ILD/low DLCO likely reflects occult pulmonary hypertension or increased pulmonary resistance that strains the RV.

From a hemodynamic perspective, the TAPSE/sPAP ratio is increasingly recognized as a non-invasive marker of RV-PA coupling and an important determinant of prognosis in pulmonary vascular disease. Grimaldi et al. recently demonstrated that a decreased TAPSE/sPAP ratio predicts adverse outcomes and is associated with elevated natriuretic peptide levels (TAPSE/sPAP ratio and NT-proANP in SSc) in SSc patients with cardiovascular involvement (19). In our cohort, TAPSE/sPAP was significantly lower in SSc patients than in controls, consistent with impaired ventriculoarterial coupling. Although TAPSE/sPAP retained independent statistical significance in the multivariate model after inclusion of MPI, it showed moderate discriminatory ability (AUC 0.71) in ROC analysis and a strong inverse correlation with PASP. These findings suggest that RV MPI encompasses a broader spectrum of RV dysfunction (including both systolic and diastolic components), while TAPSE/sPAP may better reflect the interaction between RV contractility and afterload. Taken together, the combined assessment of RV MPI and TAPSE/sPAP may provide complementary insights into the pathophysiology of RV dysfunction in SSc.

Over time, RV structural remodeling occurs in response to increased afterload, including RV free wall thickening, which was identified by Karna et al. as a key predictor of elevated MPI (9). Lindqvist et al. similarly noted that RV wall thickening is an early marker of SSc heart involvement and is likely a compensatory response to intermittent pulmonary pressure spikes (20). Thus, the interplay between myocardial fibrosis and pulmonary vascular disease creates a vicious cycle: fibrosis stiffens the heart and raises MPI, and pulmonary hypertension overloads the RV, further worsening MPI. In support of this, several studies in connective tissue diseases have reported positive associations between PASP and MPI (14). Nevertheless, the observation that some patients with high MPI had only modestly elevated PASP reinforces the role of intrinsic myocardial involvement beyond pure pressure overload. This is consistent with previous findings that patients with SSc may exhibit impaired RV diastolic function even in the absence of resting pulmonary hypertension, likely reflecting early myocardial fibrosis and latent vascular dysfunction (9, 21, 22).

Finally, SSc-related autonomic dysfunction and small-vessel vasospasm may transiently impair myocardial perfusion and contribute to cardiac dysfunction. Taken together, these mechanisms explain why RV MPI is a sensitive indicator; it increases whenever there is any combination of prolonged isovolumic times and shortened ejection time, which is exactly what happens in an SSc heart under strain from fibrosis or an elevated afterload. Importantly, these changes often precede clinical symptoms, highlighting the need to vigilantly screen patients with SSc for cardiac involvement, even if they are asymptomatic.

### Future directions and research perspectives

Our study showed that RV MPI can serve as an early marker of cardiac involvement in patients with SSc. Future research should focus on longitudinal studies to determine the prognostic value of the MPI. Following SSc patients with serial MPI measurements could reveal whether abnormal MPI values predict PAH, right-sided heart failure, or arrhythmias. Patients with SSc and high baseline MPI levels should be evaluated for worse outcomes to validate the MPI as a risk predictor.

Therefore, the integration of MPI with other cardiac biomarkers should be explored. Natriuretic peptides and cardiac troponins are recognized as biomarkers of SSc. Elevated NT-proBNP and troponin T levels occur in patients with SSc without heart failure and predict the development of PAH and poor survival. Combining MPI with serum markers could enhance detection; patients with elevated RV MPI and abnormal NT-proBNP levels could be classified as high-risk, prompting earlier intervention.

The correlation of RV MPI with cardiac magnetic resonance (CMR) imaging can enhance its utility. CMR with late gadolinium enhancement quantifies myocardial fibrosis, and CMR fibrosis correlates with worse cardiovascular outcomes in SSc. Investigating whether an elevated RV MPI correlates with higher fibrosis could refine risk prediction. Additionally, the integration of speckle-tracking strain echocardiography with MPI is valuable. Speckle-tracking strain detected early ventricular dysfunction and showed reduced RV free-wall strain in SSc patients without PAH. A combined approach using MPI with tissue Doppler and 2D strain can optimize RV dysfunction detection.

Interventional studies could assess whether early treatment normalizes the elevated RV MPI. The integration of RV MPI into SSc care shows promise as an index that, when combined with biomarkers and imaging, could improve the detection of cardiac involvement. Given the contribution of cardiac causes to SSc mortality, early identification is crucial. These findings support the routine use of cardiovascular assessments, including RV MPI, in SSc patients.

### Study limitations

There are many restrictions on this research. Initially, it was carried out at a single tertiary facility with a small sample size, which would restrict how broadly our results can be applied to other facilities. Although the study cohort was well-characterized and adequately powered to detect significant differences in RV MPI, larger multicenter studies are needed to validate these results across diverse populations. Second, the cross-sectional design precludes any assessment of longitudinal changes or causal inference; therefore, we were unable to evaluate whether an elevated MPI predicts the development of overt cardiopulmonary complications over time.

Certain advanced echocardiographic parameters, such as RV free wall strain and tissue Doppler imaging indices, were not systematically collected, and biochemical markers of cardiac stress were unavailable. Standard left and right ventricular measurements, including LVEDD, LVESD, LV mass index, LV diastolic indices, RV fractional area change, tricuspid systolic velocity (s’), and RV E/e’, were not systematically recorded, limiting echocardiographic comparisons. Although immunosuppressive treatment status was recorded, detailed data on treatment duration and changes were unavailable, precluding assessment of therapeutic effects on RV MPI. The integration of these parameters could provide insight into pathophysiological mechanisms underlying elevated MPI.

Furthermore, intra-rater and inter-rater reliability analyses (e.g., intraclass correlation coefficients) for the echocardiographic measurements were not performed, which is an additional methodological limitation. Future studies should incorporate predefined repeatability assessments to strengthen the robustness of echocardiographic indices in SSc.

## Conclusion

We showed in this cross-sectional research that RV MPI was significantly elevated in patients with systemic sclerosis compared to healthy controls, despite preserved left ventricular function. RV MPI was notably higher in patients with interstitial lung disease, reduced DLCO, and extensive skin involvement, suggesting a relationship between global right ventricular dysfunction and end-organ manifestations of SSc. Importantly, RV MPI did not correlate with inflammatory markers, disease duration, or autoantibody profile, indicating that cardiac involvement may progress independently of systemic disease activity or duration of disease.

In addition to the MPI findings, SSc patients demonstrated significantly lower TAPSE and TAPSE/sPAP ratios compared with controls, indicating early impairment of longitudinal RV systolic function and RV–pulmonary arterial coupling. In multivariable analysis, RV MPI remained independently associated with SSc status after adjusting for age and TAPSE/sPAP and showed excellent discriminative performance in ROC analysis (AUC 0.92). These results suggest that RV MPI and TAPSE/sPAP capture complementary aspects of RV dysfunction and afterload mismatch in SSc.

These findings support the use of RV MPI as a sensitive, non-invasive echocardiographic parameter for detecting early right ventricular dysfunction in patients with SSc, even in asymptomatic patients. Routine assessment of RV MPI, alongside standard RV indices and TAPSE/sPAP, may enhance risk stratification and identify candidates for closer cardiopulmonary surveillance. Future longitudinal studies are warranted to evaluate the prognostic implications of an elevated MPI and explore its integration with biomarkers and advanced imaging modalities. Overall, RV MPI appears to be a valuable adjunctive tool in the comprehensive cardiovascular assessment of systemic sclerosis.

## Data Availability

The original contributions presented in this study are included in this article/supplementary material, further inquiries can be directed to the corresponding author.
